# Cardiac Imaging in Athlete’s Heart: The Role of the Radiologist

**DOI:** 10.3390/medicina57050455

**Published:** 2021-05-07

**Authors:** Marco Fogante, Giacomo Agliata, Maria Chiara Basile, Paolo Compagnucci, Giovanni Volpato, Umberto Falanga, Giulia Stronati, Federico Guerra, Davide Vignale, Antonio Esposito, Antonio Dello Russo, Michela Casella, Andrea Giovagnoni

**Affiliations:** 1 Department of Clinical, Special and Dental Sciences, University Hospital “Ospedali Riuniti Umberto I-Lancisi-Salesi”, 60126 Ancona, Italy; agliata.g@gmail.com (G.A.); kiara.basile@hotmail.it (M.C.B.); m.casella@staff.univpm.it (M.C.); a.giovagnoni@staff.univpm.it (A.G.); 2 Department of Biomedical Science and Public Health, University Hospital “Ospedali Riuniti Umberto I-Lancisi-Salesi”, 60126 Ancona, Italy; paolocompagnucci1@gmail.com (P.C.); giovol@live.it (G.V.); u.falanga@pm.univpm.it (U.F.); giulia.emily.stronati@gmail.com (G.S.); P005471@staff.univpm.it (F.G.); a.dellorusso@staff.univpm.it (A.D.R.); 3 Department of Radiology, University Hospital “San Raffaele Hospital”, 20132 Milan, Italy; vignale.davide@hsr.it (D.V.); esposito.antonio@unisr.it (A.E.)

**Keywords:** athlete’s heart, cardiac magnetic resonance, cardiac computed tomography, cardiomyopathy, sudden cardiac death

## Abstract

Athlete’s heart (AH) is the result of morphological and functional cardiac modifications due to long-lasting athletic training. Athletes can develop very marked structural myocardial changes, which may simulate or cover unknown cardiomyopathies. The differential diagnosis between AH and cardiomyopathy is necessary to prevent the risk of catastrophic events, such as sudden cardiac death, but it can be a challenging task. The improvement of the imaging modalities and the introduction of the new technologies in cardiac magnetic resonance (CMR) and cardiac computed tomography (CCT) can allow overcoming this challenge. Therefore, the radiologist, specialized in cardiac imaging, could have a pivotal role in the differential diagnosis between structural adaptative changes observed in the AH and pathological anomalies of cardiomyopathies. In this review, we summarize the main CMR and CCT techniques to evaluate the cardiac morphology, function, and tissue characterization, and we analyze the imaging features of the AH and the key differences with the main cardiomyopathies.

## 1. Introduction

Physical exercise is useful to prevent many cardiovascular diseases, limiting several risk factors for heart pathologies and reducing the incidence of fatal ischemic events due to coronary artery disease [1]. Athletes commonly carry out intensive and excessive physical activity much more than the recommended usual quantity of healthy subjects. For this reason, athletes have a 2.4 to 4.5 increased risk of sudden cardiac death (SCD) and about 80% are asymptomatic before death [2]. Most SCDs occur, due to unknown underlying cardiomyopathies, during or just after physical activity, suggesting that multiple stress factors, such as dehydration, electrolyte, and acid-base alterations may act as a trigger [1,2,3].

Long-lasting athletic training causes cardiac heart overload and it results in morphological and functional cardiovascular adaptations of cardiac chambers, called athlete’s heart (AH). These adaptive modifications are physiological responses to the hemodynamic demands of increased cardiac output but, frequently, overlap with cardiac diseases and the differential diagnosis between AH and cardiomyopathies is a challenging task [4,5,6].

The clinical evaluation, the electrocardiogram (ECG), and echocardiography are the first-line exams that should be used to detect cardiovascular abnormalities. However, when the results are not conclusive, the cardiologist’s dilemma is often between causing unnecessary disqualification or risking a preventable catastrophic event [7].

The radiologist, specialized in cardiac imaging, could represent an essential support in this clinical decision. Cardiac magnetic resonance allows accurate and reproducible measurements of cardiac anatomy and function and, above all, it provides tissue characterization, that is pivotal for the differential diagnosis between AH and cardiomyopathy. Likewise, cardiac computed tomography (CCT) provides morphological and functional heart evaluation with low radiation dose using the last generation CT scanner. Moreover, the recent technological advances, such as the introduction of dual energy (DE) technology, enabled to extend the CCT application for the myocardial tissue characterization [7,8].

In this work, the Section 1 summarizes the role of CMR and CCT in the evaluation of myocardial morphology, function, and tissue characterization, the Section 2 analyzes the CMR and CCT imaging features of the AH and the key differences with the main cardiomyopathies.

## 2. Cardiac Magnetic Resonance Imaging

### 2.1. The Added Role

CMR is a non-radiation imaging modality with high spatial and temporal resolution. CMR is useful to evaluate the morphological cardiac findings when the echocardiography alone is not enough, because CMR has no blind spots and it does not have the same limitations, related to the presence of thoracic wall or pulmonary parenchyma or inaccurate wall thickness evaluation, due to short-axis obliquity. Moreover, CMR allows calculating accurately the global cardiac function and evaluating the regional wall motion abnormalities because it provides full ventricle coverage in all cardiac planes, such as short-axis, four-chambers, and two-chambers. Finally, the main added role of CMR is to characterize the myocardial tissue, using different techniques, such as mapping, extracellular volume images (EVC), and late gadolinium enhancement (LGE) [9].

### 2.2. Morphological Assessment

CMR is the reference standard modality to calculate the left ventricular (LV) and the right ventricular (RV) volume and mass. In clinical routine, standard evaluations include the absolute and indexes values of the LV and RV end-diastolic volume (EDV), end-systolic volume (ESV), mass, and wall thickness. The normal ranges are related to sex and age. Due to the high accuracy and reproducibility, these parameters can be used both for diagnosis and follow-up [10].

### 2.3. Functional Assessment

#### 2.3.1. Global Contractile Function

CMR is the gold standard modality to quantify the global contractile function. In the clinical routine, the evaluations include the LV and the RV stroke volume (SV), cardiac output (CO), and ejection fraction (EF). Recently, using the volume–time curve, other functional ventricular parameters have been introduced, such as the peak ejection rate, the early peak filling rate (PFRE), the peak filling rate at atrial contraction (PFRA), their ratios (PFRE/PFRA), and the values indexed to the EDV (PFRE/EDV, PFRA/EDV). Moreover through atrial volume–time curve can be calculated atrial EDV and ESV and atrial EF [11].

#### 2.3.2. Regional Contractile Function

CMR, with use of fast-cine MR sequences, allows evaluation of the regional myocardial kinetic through the subjective evaluation of the systolic-to-diastolic wall thickness changes. The main pathological wall kinetic abnormalities are the hypokinesia, defined as a decreased wall motion, the akinesia, characterized by absent wall motion, and the dyskinesia, described as uncoordinated wall motion [12].

The most recent parameter, introduced to evaluate the myocardial regional function, is the myocardial strain. It defines the degree of deformation of a fixed myocardial point throughout the cardiac cycle, from the systolic to the diastolic phases [13]. The reference modality to analyze ventricular longitudinal, radial, and circumferential strains and strain rates is cine tagging imaging. It is based on fast-cine MR sequences and allows the objective evaluation of strains without additional sequences and measurements. The cardiac regions (basal, midmyocardial, and apical) and segments (endocardial, mesocardial, and epicardial) have different strains and strain rates. The main limitation in the clinical use of strains and strain rates is that, differently to the global function parameters, the values depend on the type of MR scanner and on the specific evaluation software [14].

### 2.4. Mapping

Mapping is an MR imaging technique that allows the pixel-by-pixel estimation of magnetic relaxation times [15].

#### 2.4.1. T1 Mapping

T1 mapping technique is mainly used in the characterization changes in myocardial tissue composition in various cardiac pathologies, such as myocardial infarction, myocarditis, sarcoidosis, amyloidosis, and overload pathologies [11].

The acquisition consists of multiple T1-weighted images with different longitudinal relaxation times (T1 values), following either an inversion or saturation preparation. Then, T1 signal intensities at each pixel can be determined using a dedicated equation and T1 mapping images are generated. The main protocols applied are the modified looklocker inversion recovery (MOLLI) for inversion-recovery-based technique and saturation recovery single-shot acquisition (SASHA) for saturation-based technique. [11].

T1 mapping allows a complete myocardial evaluation and it is particularly useful in diffuse myocardial disease. The main benefit of T1 mapping is to quantify the tissue abnormalities to track myocardial changes over time and to control the disease evolution during pharmacological treatments. The main limitation is that T1 values depend on many factors, such as pulse sequence, constructor, cardiac cycle, and magnetic field, and are influenced by age [13].

T1 mapping values increase weakly with myocardial fibrosis and strongly with amyloid and myocardial edema and fall with fatty infiltration and iron overload [11,15].

#### 2.4.2. T2 Mapping

T2 mapping technique is mainly used to identify and quantify myocardial edema in different cardiac diseases including myocarditis, myocardial infarction, sarcoidosis, and amyloidosis [16].

The acquisitions are obtained in a single expiratory apnea, during the same phase of the cardiac cycle, and in successive consecutive heartbeats, every 2 to 4 RR intervals, according to the heart rate. In each slice, three T2-weighted images are obtained with different T2 preparation times, usually at 0 ms, 25 ms, and 55 ms. Then, the images are processed applying a motion correction algorithm to reduce respiratory and cardiac motion artifacts. Finally, the T2 mapping images are generated [16].

T2 mapping has several advantages compared to T2-weighted spin-echo inversion recovery sequences, traditionally used to evaluate myocardial edema, which are often of limited value due to susceptibility or slow-motion artefacts. Moreover, T2 mapping allows the quantification of edema [16].

In the absence of iron overload, T2 mapping values increase only with edema. With iron overload or myocardial hemorrhage T2 mapping values decrease [16].

### 2.5. Extracellular Volume Fraction

Myocardial ECV refers to the volume of the myocardium, which is not occupied by cells, and it is a marker of interstitial disease. This parameter allows the quantification of the myocardial extracellular volume, expressed as a percentage of myocardial mass [17].

The ECV evaluation is based on the distribution of the contrast agent in the myocardium and its calculation exploits the characteristics of the paramagnetic contrast agent, which is intravascular, extracellular, and interstitial. Considering the variation of the T1 relaxation time of the myocardium and the blood before and after contrast administration and using the hematocrit (Hct) value, the ECV estimation is obtained: ECV = (1 − Hct) × (∆R_m_/∆R_b_), where ∆R_m_ and ∆R_b_ represent, respectively, the change in T1 relaxation time in the myocardial muscle and in the blood before and after contrast administration. The delay acquisition is usually performed 10′ after contrast agent injection. In this way, it is possible to obtain an estimation of extracellular matrix, the space within the myocardium, without intracellular and intravascular compartments [18,19].

ECV increases with myocardial fibrosis, edema, amyloid, and necrosis. ECV decreases with lipomatous metaplasia [18,19].

### 2.6. Late Gadolinium Enhancement

LGE is based on the kinetic characteristics of paramagnetic gadolinium-based contrast agent that accumulates in areas of scar because the myocardial fibrosis washes out slower compared to the healthy myocardium. LGE allows to delineate the extent of fibrosis and it has a higher correlation with the histologic evaluation [20,21]. The position of LGE can help to detect the type of cardiac pathologies. The extent of LGE correlates with the severity of the phenotype and poorer outcomes. In particular, numerous recent studies have demonstrated an association between the extent of LGE and SCD. An LGE extension > 15% of LV mass is an independent risk factor for SCD, even in the absence of other conventional additional risk markers [20,21].

### 2.7. Stress Imaging

Stress CMR-imaging technique is used to identify a reduced cardiac functional reserve and to diagnose the presence of an early-stage cardiomyopathy. In athletes, this possibility is very important, especially when the pathological changes due to the underlying initial cardiomyopathy are not evident at rest. In clinical practice, guidelines suggest the use of stress CMR-imaging to differentiate the AH and cardiomyopathy when resting functional values are mild abnormal [22].

Stress CMR-imaging can be achieved by drugs or exercise; the latter is usually applied in athletes and it has several advantages. The first advantage is to avoid the use of drugs and the possible related side effects. Secondly, it reproduces the physiological activation of the cardiovascular system during exercise, allowing evaluation of not only the regional wall movement abnormalities but also the perfusion defects. The third advantage is to correlate the symptoms experienced by the athlete and the results of stress imaging. Finally, the fourth benefit is to assess the cardiac function at each stage of activity providing a unique opportunity to characterize and to differentiate exercise profiles between individuals [23].

However, stress CMR-imaging has some disadvantages. Firstly, it is necessary to adopt specific and scarcely widespread MR-safe equipment. Secondly, the motion artifacts during physical exercise can significantly reduce the overall image accuracy. To overcome these limitations, it has been proposed to acquire images after the temporary interruption of maximum exercise with the treadmill positioned outside the scanning room. However, not all movement abnormalities persist during the recovery phase, and this approach risks obtaining a high number of false negative results. With the MR-safe equipment, physical exercise can be carried out inside the scanning room allowing a significant reduction of the time between the interruption of the exercise and the start of the exam. In addition, cyclo-ergometers connected to the scanning table can allow the physical activity in supine potion in the magnet bore, and real-time imaging can be performed [24].

Therefore, with the recent introduction of rapid CMR sequences and the availability of CMR-safe equipment, stress imaging is moving from being a research field to use in clinical practice. However, despite these results, further studies are necessary to evaluate the cost-effectiveness of stress CMR-imaging to distinguish the early-stage cardiomyopathies and AH [25].

## 3. Cardiac-CT

### 3.1. The Added Role

The introduction of technological advances enabled the extended use of CCT beyond coronary evaluation. New CT scanners allow high spatial and temporal resolution, and CCT can be used to obtain high-quality multiplanar reconstructions in any desired image orientation with low contrast volumes and low radiation dose. It is a viable alternative modality to CMR to evaluate morphological parameters and global and regional kinetic functions. Moreover, the use of DE technology provides the tissue characterization with the evaluation of ECV and late iodine enhancement (LIE) [26,27].

The main CCT advantages compared with CMR are the feasibility and short examination time. Therefore, CCT is particularly useful in patients with relative and absolute contraindications for CMR or in the case of claustrophobic patients, which may be unable to undergo the examination [27,28].

### 3.2. Morphological and Functional Assessment

CCT allows the evaluation of the main cardiac morphological parameters, such as volume, mass, and wall thickness. The values demonstrated excellent reproducibility and a very good linear relationship compared to those obtained with CMR, even if the normal CCT values are significantly lower than those obtained with CMR [28]. The knowledge of the normal ranges is essential for the differential diagnosis between AH and cardiomyopathy. Normal values of LVEDV and LVESV were, respectively, 112.9 ± 26.1 mL and 41.7 ± 14.7 mL, the normal value of LV mass was 145.0 ± 29.1, and the normal values of the septal and posterior wall thicknesses were, respectively, 1.08 ± 0.18 cm and 0.91 ± 0.15 cm [8,28].

Moreover, CCT provides global and regional functional analysis. Many prior studies validated, via comparisons with CMR, the global functional parameters, such as SV, CO, and EF, and the evaluation of regional functional findings, such as hypokinesia, akinesia, and dyskinesia [29].

Morphological and functional findings are comprehensively assessed using a retrospective ECG-gated helical scanning protocol, which is characterized by continuous data acquisition throughout some consecutive cardiac cycles. This retrospective technique allows the evaluation of any cardiac phase and it is particularly useful in patients with high or irregular heart rhythm and, even if it is associated with relatively high radiation doses, it allows with the same acquisition the evaluation of the coronary artery disease [8,29].

### 3.3. Extracellular Volume Fraction

Myocardial ECV provides a remodeling value of the cardiac tissues and offers a measure of the proportion of the extracellular space within the total mass of the myocardium. ECV is a reproducible index and it demonstrated good agreement with the same value calculated with CMR. Indeed, iodine-based contrast medium has similar pharmacokinetic characteristics compared to the paramagnetic contrast agent [8,30].

ECV is calculated with the formula: ECV = (1 − Htc) × (ΔHU LV_m_/ΔHU_b_), where ΔHU LV_m_ and ΔHU_b_ represent the Hounsfield Unit difference between the pre-contrast and the post-contrast phase, respectively, in the myocardium and in the blood. The post-contrast phase is usually performed 5–7 min after contrast injection [8,30].

### 3.4. Late Iodine Enhancement

LIE is used to evaluate myocardial fibrosis and it is an alternative modality to identify myocardial scar when CMR or gadolinium are contraindicated. LIE demonstrated good reproducibility and excellent correlation with LGE. In particular, the scar regions show a slow washout of iodine-based contrast medium and appear hyperdense in the delayed phase compared with the healthy myocardium. The delayed post-contrast phase is usually performed from 7 to 10 min after the contrast administration [31,32].

The use of DE technology is strongly recommended to evaluate LGE. This technology used two different tube voltages (kV), usually a low (from 70 to 90 kV) and a high (from 140 to 150 kV) voltage. This acquisition allows generation of monoenergetic images with a high contrast-to-noise ratio to detect LIE. Different DE technologies can be used, such as the rapid switching of X-ray tube potential, the multilayer detector, or dual X-ray tubes. The total volume of the iodine contrast medium depends on the contrast concentration and on the patient’s weight, and usually, the range is from 1.2 mL/kg to 1.5 mL/kg [31,32].

## 4. Athlete’s Heart

### 4.1. Definition

AH is characterized by all the cardiac adaptations related to physical training. These cardiac changes are due to systemic arterial resistance reduction and volume overload. Therefore, the morphological modifications are the increased cardiac volumes and the myocardial hypertrophy. The main functional adaptation is an augmented stroke volume with a usually normal or slightly reduced EF at rest, showing a good contractile reserve [1,2,6].

**Figure 1:**
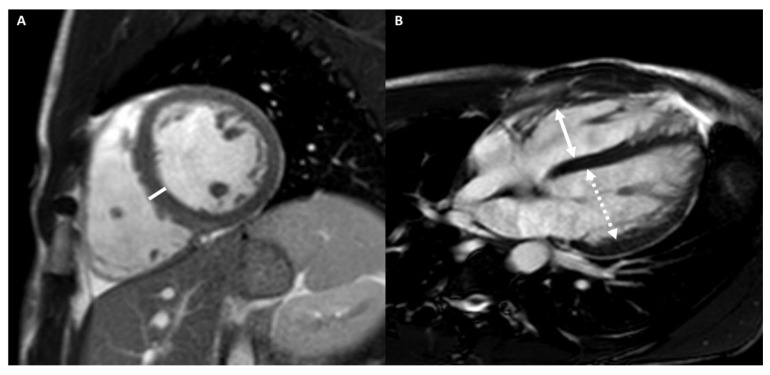

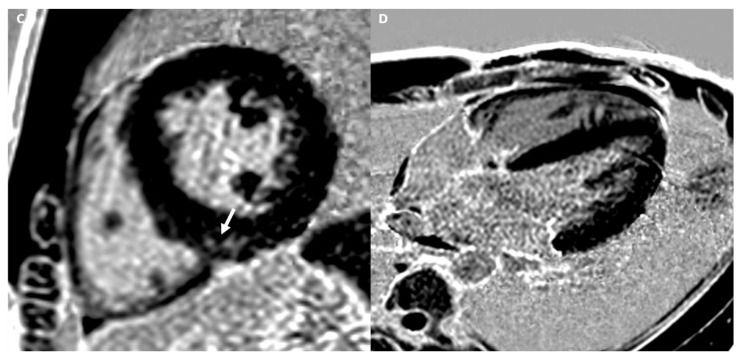
A 20-year-old male athlete (runner) with palpitation. Cine-CMR images show, in short-axis (**A**) and in four-chamber (**B**) planes, LV symmetrical eccentrical hypertrophy (13 mm) (line), bi-ventricular dilations with LV maximum diameter of 60 mm (dashed double-headed arrows) and RV maximum diameter of 56 mm (full double-headed arrows) and mid-apical bi-ventricular hyper-trabeculation. Delay-CMR contrast images demonstrate, in short-axis (**C**) plane, a linear LGE in the mesocardial layer at the postero-septal junction (arrow), instead, no LGE areas are visible in four-chamber plane (**D**). The final diagnosis was physiological cardiac modifications in AH.

Figure 1 shows an example of a subject with AH.

### 4.2. Differential Diagnosis

Intense prolonged training can induce cardiac changes that overlap with cardiomyopathy and occasionally athletes may die suddenly during training or competition. For this reason, it is essential to distinguish athletic cardiac remodeling and cardiomyopathies [2,6].

Table 1 summarizes the main differences in imaging features between AH and cardiomyopathies in CMR and CCT.

#### 4.2.1. Cardiac Imaging: AH vs. Hypertrophic Cardiomyopathy

Hypertrophic cardiomyopathy (HCM) is a non-ischemic cardiac disease, characterized by a wall thickness ≥ 15 mm at ED, in at least one LV myocardial segment, or a ratio between septal thickness and inferior wall at mid-ventricular level >1.3, without pressure overload or infiltration. LV wall thickness ≥ 30 mm is associated with SCD [33].

The evidence of pathological ECG changes, particularly abnormal T wave inversion, without echocardiogram anomalies represent the main indication for the CMR or CCT examinations. CMR or CCT can identify the apical HCM and can precisely measure the extension and the pattern of LV wall thickness, especially in segments less reliably visualized by echocardiography when the acoustic window is poor. Furthermore, they can provide information about myocardial fibrosis and they can reveal features, such as papillary muscle hypertrophy, basal-apical muscle bundle, and clefts [34,35].

Athletes who train regularly may have physiological LV hypertrophy, which can be difficult to differentiate from HCM [9,10].

Sports with a high-static component cause a concentric hypertrophy with an increased ventricle mass without chamber dilation. Sports with high-dynamic components determine an eccentric hypertrophy with an increase in ventricle mass and size. In AH, LV hypertrophy is usually symmetrical. The main morphological findings that support the diagnosis of HCM are the presence of an asymmetric apical or septal hypertrophy, a value of maximum diastolic thickness/minimum diastolic thickness ≥ 1.3, a value of LV diastolic wall-to-volume ratio ≥ 0.15 mm/m^2^/mL, and an increased number of papillary muscles, that are usually displaced and hypertrophic (> 11 mm). Other typical secondary signs of HCM are myocardial crypts, para-septal muscle bundle, and abnormal apical trabeculation. Moreover, AH hypertrophy regresses after a rest detraining period. Indeed, a reduction greater than 2 mm, in the absence of exercise over a three-month period, supports the diagnosis of AH, while an unchanged hypertrophy suggests the diagnosis of HCM [9,10].

In AH, global and regional functions are preserved. HCM, due to structural alterations such as fibrosis and the disarray of myocardial fibers, is usually characterized by a diastolic dysfunction, with normal or supranormal SV and EF. Finally, strains and strain rate can be mildly reduced in HCM, instead, in AH, they are preserved [16,20].

Mapping and ECV can be used to differentiate between AH and HCM. Indeed, in AH, hypertrophy is due to a percentage increase in the cellular component and a relative reduction of the extracellular space, on the contrary in HCM, the hypertrophic component is due to the presence of myocardial fibrosis, which causes a relative higher extracellular component and a percentage reduction of the cellular space. Hence, T1 mapping and ECV values are reduced in AH and increased in HCM. T1 value > 1217 ms and ECV value > 22.5% have the greatest sensitivity and specificity for the diagnosis of HCM [4,9,10]. Therefore, T1 mapping and ECV could be used for the differential diagnosis between AH and HCM when the wall thickness reaches values close to the pathology threshold, between 12 to 15 mm [2,36].

LGE is normally absent in AH and when present it appears as a mesocardial stria located at the anterior and/or posterior interventricular junctions and at the insertion point of the trabeculae on the ventricular wall [37]. The main cause of the presence of LGE appears to be related to the high systolic pressure induced by exercise. Indeed, a correlation between training intensity and LGE has been demonstrated. The clinical relevance of LGE is still poorly understood and the management of athletes with cardiac fibrosis is still unclear [8,9]. However, as stated, the presence of fibrosis in the AH is rare and the detection of LGE, shifts the diagnosis away from the AH, in favor of HCM. Indeed, up to 60% of HCM have LGE, which usually is an irregular or massive area located in the site of greatest wall thickness associated with mesocardial striae at the anterior and posterior interventricular junctions [8,9].

Although the detection of LGE favors the diagnosis of HCM, the absence does not exclude the possibility of HCM. For these reasons, considering the actual clinical evidence, even if current guidelines recommend the only fibrosis evaluation, a comprehensive CMR tissue characterization that comprises mapping, ECV and LGE should be performed in every athlete with suspected HCM [8,9].

Particularly when CMR is contraindicated, the evaluation with CCT could be considered. CCT provides anatomical information about HCM based on key morphological characteristics (presence, location, distribution, and severity of HCM), LVEF and the extent of myocardial fibrosis. As for the CMR, mean ECV values with HCM are significantly higher than in AH [4,9].

**Figure 2:**
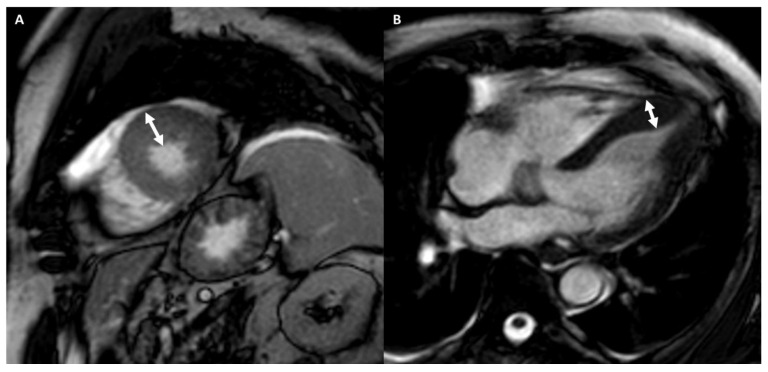

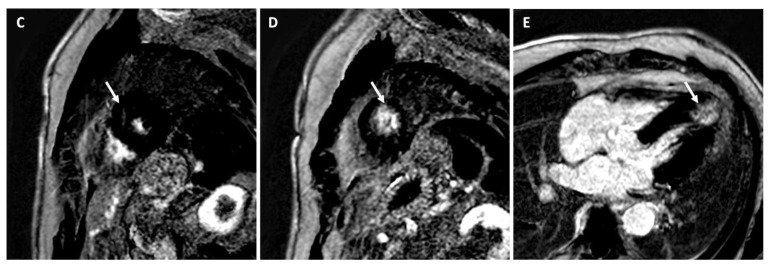
A 25-year-old male athlete (soccer player) with ECG anomalies. Cine-CMR images show, in short-axis (**A**) and in four-chamber (**B**) planes, LV asymmetric apical hypertrophy with maximum thickness at the level of antero-septal junction (22 mm) (double-headed arrows). Delay-CMR contrast images demonstrate the presence of linear mid-wall LGE (arrow) at the level of antero-septal junction (**C**) and massive LGE at the hypertrophic apical level in short-axis (**D**) and in four-chamber (**E**) planes. The final diagnosis was HCM.

**Figure 3:**
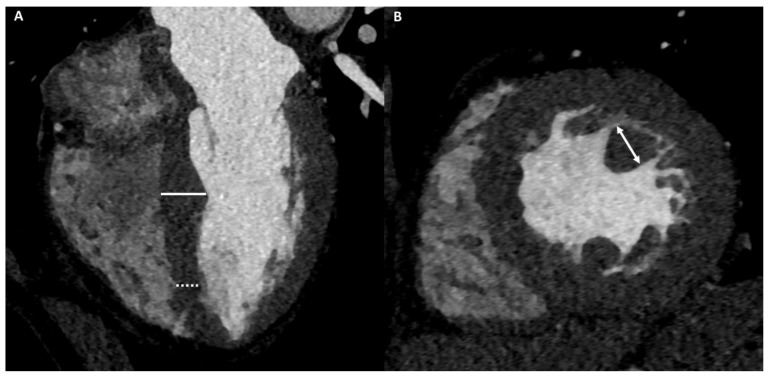

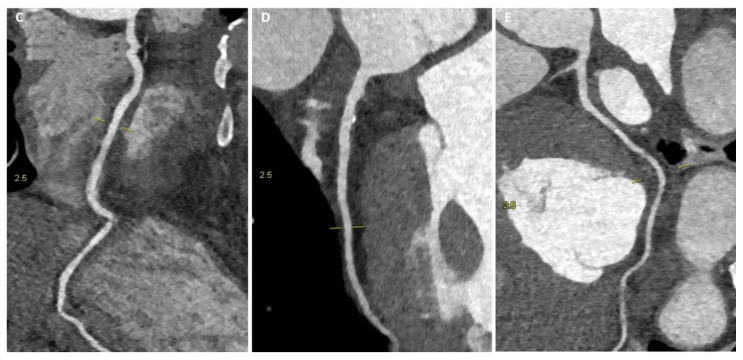
A 36-year-old male athlete (soccer player) with syncopal episode. CCT images show, in four-chamber plane (**A**), asymmetric septal hypertrophy with maximum diastolic thickness (16 mm) (full line) and minimum diastolic thickness (11 mm) (dashed line) and, in short-axis plane (**B**), a hypertrophic papillary muscle (14 mm) (double-headed arrows). With the same scan acquisition, significant stenosis was excluded in the right coronary artery (**C**), in the anterior descending artery (**D**), and in the circumflex artery (**E**).

Figure 2 and Figure 3 show the examples of two athletes with HCM evaluated, respectively, with CMR and CCT.

#### 4.2.2. Cardiac Imaging: AH vs. Dilated Cardiomyopathy

Dilated cardiomyopathy (DCM) is a non-ischemic heart muscle disease. It is defined as dilation of both ventricles, exceeding normal value of 112%, with EF lower than 45%, in the absence of coronary artery disease or increased load conditions. Occasionally, it is also associated with hyper-trabeculation of both ventricles [10,38].

CMR and CCT are particularly useful to evaluate the volumes and the EF in subjects with poor acoustic echocardiographic windows and to characterize the tissue composition [10,38].

The AH overlaps features of DCM [39]. About half of the athletes present bi-ventricular dilation, due to the volume overload. Sports with a high-dynamic component are associated with a higher dilation compared with sports with a prevalent static component. Indeed, up to 14% of professional athletes show ventricle diameters greater than 60 mm. In athletes engaged in endurance disciplines such as cycling, triathlon, or rowing, an LVED diameter of up to 66 mm in females and 70 mm in males was observed. The main differential finding between AH and DCM is that the first one includes the dilation of both ventricles, instead, the second one involves only the LV [39].

Detraining is a common test used to differential diagnosis between AH and DCM. Indeed, despite the limited data, in most athletes, there is a reduction in the cardiac dilation after 3 months from the cessation of training. However, adherence to detraining is poor and the identification of the AH using non-invasive imaging remains preferable [40].

Considering functional findings, AH has a slightly reduced ejection fraction (typically 45–55%), especially in sports with high-dynamic activity. In this case, the use of stress CMR-imaging may be helpful to correctly differentiate AH from DCM. Indeed, while athletes can significantly increase EF during exercise, patients with DCM less so. A cutoff value of 11.2% for the increase in LVEF from rest to peak exercise has a sensitivity of 93% and a specificity of 90% to differentiate AH from DCM. However, the role of stress CMR-imaging is not validated, and the best threshold of contractile reserve has not been well defined [39].

Mapping and ECV are useful techniques to differentiate AH and DCM. T1 mapping, T2 mapping, and ECV values are lower in athletes than in DCM, and T1 mapping seems to be the most accurate parameter to differentiate these two conditions [41].

LGE is present in about 30–50% of the patients with DCM. The most characteristic pattern is a linear LGE, located in the mesocardial layer, at the infero-lateral wall or at the interventricular septum [41].

In the diagnostic workup of DCM, CCT is recommended to exclude coronary artery disease with low pretest probability. In addition, as a one-stop-shop exam, CCT can evaluate LV global systolic dysfunction and calculate LV size, wall thickness, and LIE. Moreover, CCT allows demonstrating expansion of ECV in patients with DCM compared with healthy subjects [42,43].

**Figure 4:**
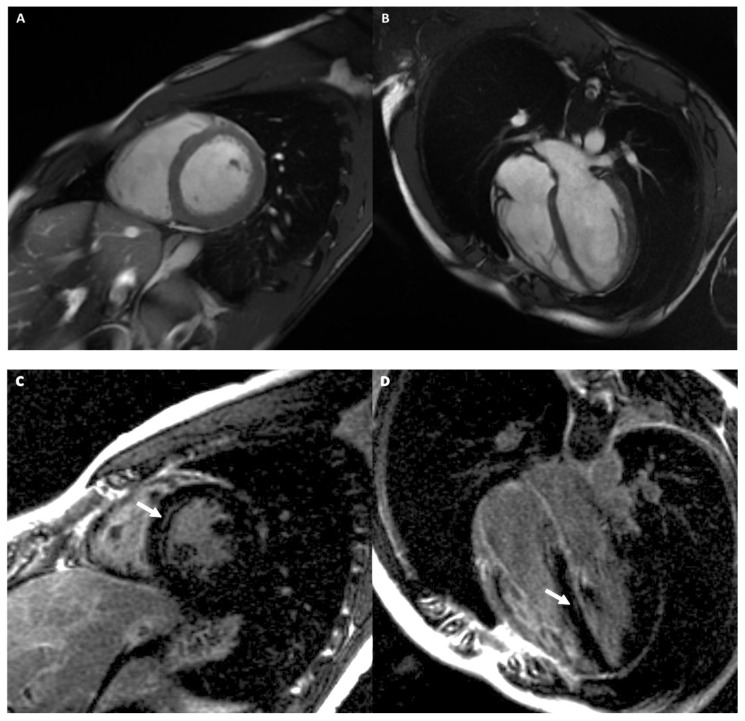
A 40-year-old male athlete (cyclist) with thoracic pain. Cine-CMR images show LV and RV dilation in short-axis (**A**) and in four-chamber (**B**) planes. Delay-MRI contrast images demonstrate, in short-axis (**C**) and in four-chamber (**D**) planes, linear mesocardial LGE at the mid-basal septum (arrows). The final diagnosis was DCM.

**Figure 5:**
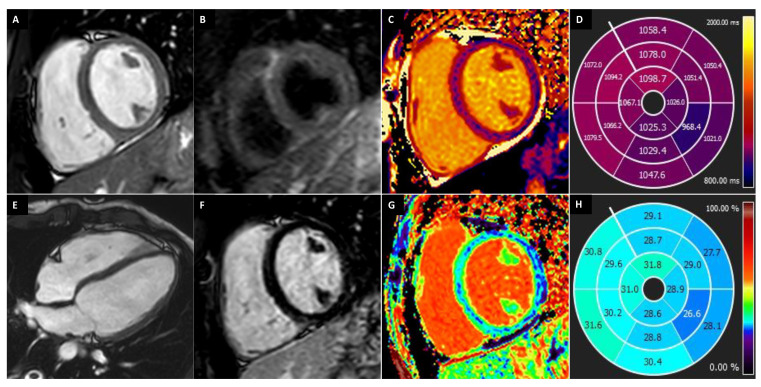
A 27-year-old female athlete (running) with ECG-Holter evidence of >15,000 premature ventricular contractions per day. Cine images show a mild enlargement of LV (**A**,**E**) with normal wall thickness and mildly reduced ejection fraction (**EF**: 52%). Short-tau inversion recovery (**B**) and LGE (**F**) sequences show unremarkable findings. T1 mapping (T1 map in (**C**), bullseye plot in (**D**)) shows slightly increased T1 values, with average septal native-T1 of 1076 ms (site specific normal values < 1045 ms). Pre- and post-contrast T1 mapping were as analyzed to generate the ECV map (**G**), which demonstrated mild increase of ECV values (bullseye plot in (**H**); average septal ECV 30.6%; normal values < 27%), suggestive of slight expansion of the myocardial interstitium. The finding of increased native-T1 and ECV values should suggest an early non-ischemic dilated cardiomyopathy or maladaptive remodeling to exercise training, rather than a physiological adaptation to training.

Figure 4 and Figure 5 show two examples of athletes with DCM.

#### 4.2.3. Cardiac Imaging: AH vs. Left Ventricular Non-Compaction

Left ventricular non-compaction (LVNC) cardiomyopathy is a myocardial phenotype that overlaps with hypertrophic and dilated cardiomyopathies and is characterized by prominent LV trabeculation [43].

Athletes, especially in high-dynamic sports, showed an increased prevalence in LV trabeculation and it is more common in African/Afro-Caribbean compared with Caucasian athletes. For this reason, the differential diagnosis between AH and LVCN can be a challenging task and it is usually over-diagnosed in athletes, particularly black athletes [2,4].

CMR and CCT are particularly useful to detect the exact extension of hyper-trabeculation and to differentiate the physiological hyper-trabeculation in AH and the pathological LVNC, usually associated with marked repolarization abnormalities and low EF [2,4].

The main finding for the differential diagnosis between hyper-trabeculation in AH and LVNC is the morphological one, which can be evaluated using different criteria. Petersen’s criteria indicate the diagnosis of non-compaction with a ratio between non-compacted (NC) myocardium and compacted (C) myocardium > 2.3, evaluated in ED, and in four-chambers plane, in at least two consecutive segments. Usually, the most frequent cardiac segments involved are the mid-apical ones of the LV inferior-lateral wall and septum. Subsequently, to improve the sensitivity and diagnostic specificity, Jacquier’s criteria were introduced, which support the diagnosis of non-compact myocardium with a ratio between NC/C mass > 20%, evaluated in ED, and in short-axis plane. Finally, Captur’s criteria suppose the diagnosis of non-compaction considers the complexity of the myocardial NC distribution based on mathematical model and fractal analysis. However, none of the criteria demonstrated adequate diagnostic accuracy and with a low pretest probability of LVNC, these values could generate false positive diagnoses [2,4,8].

Considering functional findings, LVNC, although often has slightly reduced EF, usually shows strain and strain rates reduction. This observation implies that strain myocardial alteration occurs early in the course of the disease and underlines the utility of deformation CMR-imaging for evaluating LV function compared to LVEF alone [2,4,8,44].

T1 mapping values are higher in LVNC patients compared with normal controls, even in the absence of LGE, suggesting that T1 mapping can detect myocardial fibrosis in an early stage [8].

In LVNC, LGE has usually a linear pattern localized in the mesocardial layer and does not closely correlate with the NC areas. It is interesting to underline that LGE is the only independent predictor of systolic LV impairment and it is an independent parameter of poor outcome. On the contrary, the thickness of LV trabeculation does not have a significant prognostic role [2,42].

CCT allows the detection of trabeculations along the LV wall, the precise measurement between NC and C thickness ratio, and the diagnosis of the subendocardial areas of LIE. Additional cardiac CT findings may include LV systolic dysfunction evaluation with low radiation dose protocol [2,42,43,44].

**Figure 6:**
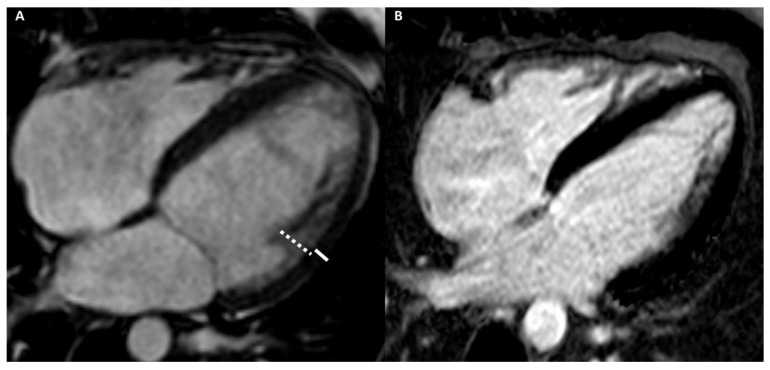
A 29-year-old male athlete (soccer player) with ECG anomalies. Cine-CMR images show hyper-trabeculation of mid-apical lateral LV wall with the non-compacted wall thickness of 13 mm (dashed line) and the compacted wall thickness of 5 mm (full line) (**A**). The ratio between non-compacted/compacted was 2.6, with the achievement of the Petersen’s criteria. Delay-MRI image demonstrates the absence of LGE (**B**). The final diagnosis was LVNC cardiomyopathy.

Figure 6 shows an example of an athlete with LVNC cardiomyopathy.

#### 4.2.4. Cardiac Imaging: AH vs. Arrhythmogenic Cardiomyopathy

Arrhythmogenic cardiomyopathy (AC) is a non-ischemic heart disease characterized by the mutation of the desmosomes, the intercellular junctions between myocardial cells. The cascade of the pathophysiological process begins with the rupture of defective desmosomes followed by apoptosis or necrosis of myocardial cells and subsequent replacement by fibro-adipose tissue. It is necessary to underline that fibro-fatty replacement does not occur homogeneously at the cardiac muscle. The disease first affects the subepicardial layer and then becomes transmural. For this reason, there are several variants of AC. The two main forms of AC are arrhythmogenic right ventricular cardiomyopathy (ARVC), the most frequent, and the atypical form, left dominant (LD) AC, which caused a high percentage of AC-related deaths [5,45].

CMR is the most suitable imaging modality to study the cardiac chambers because, compared to echocardiography, it has a higher sensibility in the identification of anatomical, functional, and tissue pathological changes, especially in early AC [46,47].

##### Arrhythmogenic Right Ventricular Cardiomyopathy

ARVC is characterized by the replacement of the RV myocardium with fibro-adipose tissue, resulting in RV dilation, dysfunction, and arrhythmia [2].

Especially at an early stage, the cardiac aspects of ARVC can overlap AH, making ARVC a challenging diagnosis in athletes. However, the differential diagnosis between physiological or pathological modification of RV is very important, because ARCV is related to up to 20% of sudden cardiac death in athletes [48].

In 2010, the criteria for the ARCV diagnosis were changed, adding the quantitative CMR-parameters. These criteria include akinesia or dyskinesia or regional dyssynchronous contraction of RV associated, in the major criteria, with RVEDV ≥ 110 mL/m^2^ (male) or ≥100 mL/m^2^ (female) or RVEF ≤ 40% and, and in the minor criteria, with RVEDV from ≥100 to <110 mL/m^2^ (male) or ≥90 to <100 mL/m^2^ (female) or RV ejection fraction from >40% to ≤45% [5,12,48].

The diagnostic criteria of ARVC in CMR are based on a combination of regional and global kinetic anomalies and anatomical alterations. However, there could be several problems in the distinction between AH and ARVC. Firstly, athletes may exhibit mild to moderate RV dilation because the thin RV wall and the propensity for a volume overload adaptation explain the earlier anatomical modifications of the RV compared to LV. Indeed, prolonged and intense physical exercise, such as occurs endurance sports, often causes moderate RV dilation. Conversely, intense and short-term efforts are associated with mild or without RV dilation. Other elements that need to be considered in evaluating the RV dilation are the athlete’s age and the number of years of training, because older athletes with more years of training show a greater degree of RV dilation. Secondly, the misdiagnosis of ARVC in athletes can occur frequently because the assessment of regional functional impairment of the RV wall is subjective and depends on the operator’s personal experience. Finally, athletes may also exhibit a mild RVEF decrease [5,12,48].

Fortunately, there are some significant differences between AH and ARVC. The first difference is that in AH, unlike ARVD, the dilation of the RV is associated with a concomitant LV volume increment, due to a global and symmetrical adaptation of the heart induced by training. For this reason, a ratio of RVEDV/LVEDV < 1.2 has been proposed to distinguish between physiological remodeling and ARVC. The second difference is that AH does not show the regional contractile RV abnormalities and has normal strain and strain rate, differently ARVC may have strain reduction. The third difference concerns the RVEF. In athletes, although there may be a mild reduction in RVEF, rarely this parameter is < 45%, as occurs in patients with ARVD [5,48,49].

Tissue characterization is not included in the CMR diagnostic criteria for ARVC. However, it may represent an important element of differential diagnosis because the presence of LGE in the free wall of the RV with morphological and functional abnormalities can be useful to support the diagnosis of ARVD. However, some possible difficulties in assessing the LGE need to be considered. The first pitfall concerns the identification. Indeed, the diagnosis of LGE in the thin RV wall may be problematic. However, notable advances in MR scanners and rapid pulse sequence updates have improved this possibility. The second pitfall is about the interpretation. Indeed, although the LV does not show dilation and dysfunction, CMR reveals a subepicardial LGE stria at the infero-lateral LV wall, up to 70% of patients with ARVD. The underlying disease process of ARVC affects the entire myocardium and left myocardial LGE can be demonstrated in ARVC patients, even in the absence of LV regional function abnormalities. For the differential diagnosis, it is essential to remember that LGE, in AH, is usually mesocardial at anterior and/or posterior interventricular junctions [48,49].

Finally, in AH, the exercise-induced remodeling of the RV is a dynamic process and can decrease or increase in relation to the training load. Conversely, in ARVC, RV dilation is irreversible [48,49].

**Figure 7:**
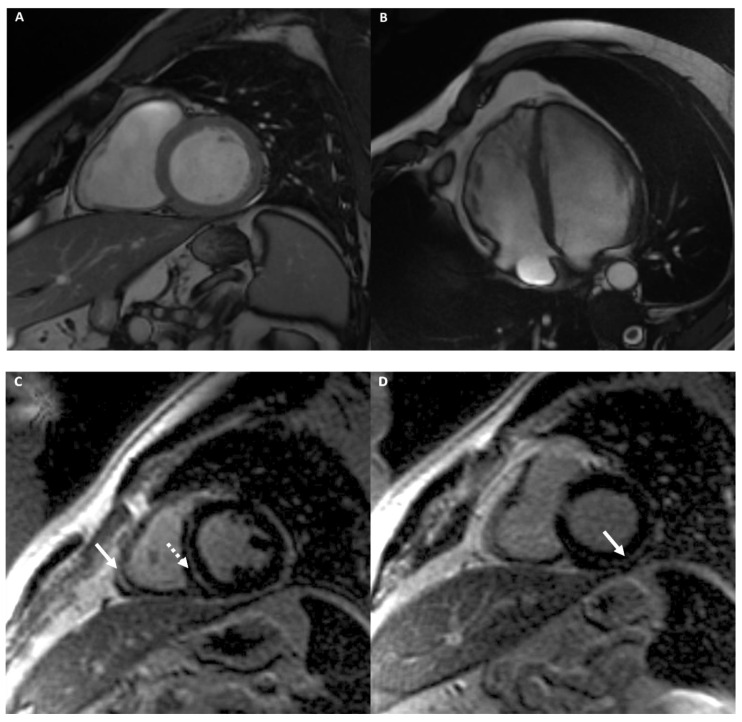
A 34-year-old male athlete (cyclist) with ECG anomalies. Cine-CMR images show RV dilation with increased dimension in short-axis (**A**) and in four-chamber (**B**) planes. Delay-MRI contrast images demonstrate, in short-axis plane (**C**), linear mesocardial LGE at RV free wall (full arrow) and at the mid septum (dashed arrow) and subepicardial infero-lateral basal LV wall (arrow) (**D**). The final diagnosis was ARVD.

Figure 7 shows an example of an athlete with ARVD.

##### Left Dominant Arrhythmogenic Cardiomyopathy (LDAC)

LDAC is characterized by mild LV dilation without RV involvement, and the fibro-fatty replacement initially concerns the subepicardial layer of the LV infero-lateral wall [50,51].

In athletes, LDAC causes ventricular arrhythmias during exercise and the probability of detection with the ECG and echocardiography is low. For this reason, the identification of myocardial fibrosis replacement may be useful [50,51].

The occurrence of exercise-induced repetitive premature ventricular beats with a right bundle-branch block may merit the cardiac evaluation with the use of CMR to rule out the subepicardial scar in the infero-lateral LV wall, which is the imaging hallmark of LDAC and may represent the only abnormal cardiac finding suggesting the differential diagnosis with AH [50,51].

**Figure 8:**
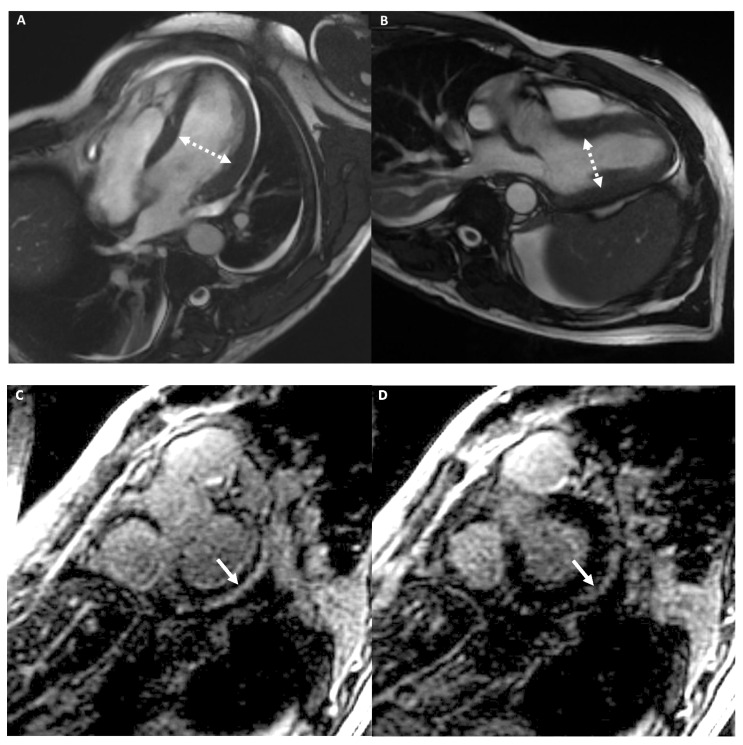
A 39-year-old male athlete (cyclist) with palpitations. Cine-CMR images show mild LV dilation with increased dimension in four-chambers (**A**) and in LV outflow-tract (**B**) plane (dashed double-headed arrows). Delay-MRI contrast images demonstrate, in short-axis planes (**C**,**D**), a subepicardial linear LGE at the basal infero-lateral LV wall (arrow). The final diagnosis was LDAC.

Figure 8 shows an example of an athlete with LDAC.

#### 4.2.5. Cardiac Imaging: AH vs. Infiltrative Cardiomyopathy

Infiltrative cardiomyopathy (IC) is a non-ischemic cardiomyopathy that includes a variety of myocardial disorders, resulting from the deposition of abnormal substances within extracellular and intracellular cardiac spaces. The main IC disorders are cardiac amyloidosis, sarcoidosis, and hemochromatosis and cause a gradual wall thickening, dysfunction up to heart failure [33].

The AH is characterized by a slight thickening of the LV myocardium and differential diagnosis with IC is required. Morphologically, IC is characterized by myocardial thickness, normal ventricular size, and atrial dilation. IC causes diastolic dysfunction, with reduction of CMR-strain values [33].

Furthermore, mapping, ECV, and LGE are crucial for the differential diagnosis. T1 mapping and ECV values are higher in cardiac amyloidosis and sarcoidosis than in AH. Differently, in hemochromatosis, T1 values are lower due to the iron deposition. LGE in cardiac amyloidosis typically has a diffuse circumferential endomyocardial distribution. Unlike in cardiac sarcoidosis, LGE has a nonspecific distribution, but usually involves the interventricular septum or the LV lateral wall and papillary muscles with a patchy mesocardial layer pattern and relative sparing of the subendocardial layer [52].

## 5. Conclusions

AH is associated with structural cardiac changes due to intense and prolonged exercise. These modifications frequently simulate those observed in cardiomyopathies that represent one of the leading causes of sudden cardiac death in athletes.

Correct differentiation between physiological AH changes and cardiomyopathies requires a comprehensive knowledge of the main imaging features and an in-depth consciousness that the cardiac adaptations are related to age, sex, ethnic characteristics, and, above all, intensity and duration of the training exercise.

In this context, the radiologist, specialized in cardiac imaging, can have a pivotal role in differentiating between AH and cardiomyopathies, as CMR and CCT are particularly useful when there are nonspecific or inconclusive findings by electrocardiography or echocardiography.

## Figures and Tables

**Table 1:** The main differences in imaging features between AH and cardiomyopathies in CMR and CCT.

	AH	HCM	DCM	LVNC Cardiomyopathy	ARVD	LDAC	IC
Morphological evaluation							
Wall thickness	<15 mm; Symmetric	>15 mm; Asymmetric	-	-	-	-	Mild increased
Volume	Increased (LV-RV)	-	Increased (LV-RV)	Increased (LV)	Increased (RV)	Mild increased (LV)	Atrial dilation
Trabeculation	Increased	Increased	Increased	NC/C > 2.3 in two cardiac segments	-	-	-
**Functional evaluation**	Normal systolic and diastolic function	Diastolic dysfunction	Systolic dysfunction	LV systolic dysfunction	RV systolic dysfunction	Mild LV systolic dysfunction	Diastolic dys-function
**Strains and strain rate**	Normal	-	-	Reduced	Reduced	-	Reduced
**Mapping**	Reduced (T1-T2)	Increased (T1)	Increased (T1-T2)	Increased (T1)	-	-	Increased (T1)
**ECV**	Reduced	Increased	Increased	-	-	-	Increased
**LGE/LIE**							
Type	Linear	Patchy or massive	Linear	Linear	Linear	Linear	Linear or parchy
Layer	Mesocardial	Mesocardial	Mesocardial	Mesocardial	Subepicardial Mesocardial	Subepicardial	Subendocardial Mesocardial
Site	Interventricular juctions	Hypertrophic area; interventricular junctions	Septum; infero-lateral LV wall	Variable, not associated with NC area	Anterior RV wall	Infero-lateral LV wall	Circumferential; septum; lateral LV wall

Abbreviations–AH: athlete’s heart; HCM: hypertrophic cardiomyopathy; DCM: dilated cardiomyopathy; LVNC: left-ventricular non-compaction; ARVD: arrhythmogenic right ventricular dilation; LDAC: left-dominant arrhythmogenic cardiomyopathy; LV: left ventricle; RV: right ventricle; NC: non-compacted; C: compacted; EF: ejection fraction; ECV: extracellular volume; LGE: late gadolinium enhancement; LIE: late iodine enhancement.

## Data Availability

Data is contained within the article.
